# P318: Safety of blood products in Rwanda

**DOI:** 10.1186/2047-2994-2-S1-P318

**Published:** 2013-06-20

**Authors:** S Florent, Maréchal Gasana

**Affiliations:** 1 Rwanda National Centre for Blood Transfusion, Kigali, Rwanda

## Introduction

Rwanda established the National Center for Blood Transfusion (NCBT) with the mission to provide safe, effective, and adequate blood and blood products to all patients in need. This is done by collecting blood exclusively from low risk VNRBD and tests each sample for TTIs by using QMS. This paper showcases NCBT operations in ensuring safe blood and blood products.

## Methods

The NCBT has implemented strategies based on QMS for NRVBD safety (questionnaire, clinical exams, etc), testing using automate machine (immuno-hematology and serology), using BECS to manage individual record, and developed national guidelines for rational use of blood in hospital, therefore extending NCBT safety procedures from vein to vein.

## Results

Routine data of NCBT shows the following: New donors (38.5%, 30.5%), irregular Donors (11.4%, 18.1%), regular donor (50.1%, 51.4%) respectively 2011 and 2012.

The serology results from initial testing shows that in 2011 among 37811 blood donors, 203 (0.54%) were HIV reactive, 609 (1.61%) HBV reactive, 1113(2.94%) HCV reactive and 668 (1.77%) were Syphilis reactive. In 2012 among 40520 blood donors recruited 203(0.50%) were HIV positive, 616 (1.52%) HBV positive, 1146(2.83%) HCV positive and 628 (1.55%) were syphilis positive.

## Conclusion

The result shows an increase of blood collected and a decrease of TTIs results in the last 2 years. This is associated to strategies implemented by NCBT on blood donor selection and quality testing through corrective and preventive action to reduce to the lowest level the risk of infectious agent.

## Disclosure of interest

None declared.

